# The Effect of Temperature and Sputtered Particles on the Wettability of Al/Al_2_O_3_

**DOI:** 10.3390/ma14092110

**Published:** 2021-04-22

**Authors:** Yang Li, Hailong Shang, Bingyang Ma, Xuqiang Guo, Rongbin Li, Geyang Li

**Affiliations:** 1 College of Engineering, China University of Petroleum-Beijing at Karamay, 355 Anding Road, Karamay 834000, China; lyanga@cupk.edu.cn (Y.L.); guoxq@cup.edu.cn (X.G.); 2 School of Materials, Shanghai Dianji University, Shanghai 200240, China; shanghl@sdju.edu.cn (H.S.); lirb@sdju.edu.cn (R.L.); 3 State Key Laboratory of Metal Matrix Composites, Shanghai Jiao Tong University, Shanghai 200240, China; gyli@sdju.edu.cn

**Keywords:** wettability, interface strength, sputtered film, Al_2_O_3_ ceramic, aluminum

## Abstract

Two kinds of Al_2_O_3_ ceramic samples with and without Al film deposited were designed respectively. The influences of temperature and high kinetic energy sputtering particles on the wettability and interface strength of Al/Al_2_O_3_ were studied by comparing the wetting behavior of molten aluminum on two samples. The results show that molten aluminum does not wet the Al_2_O_3_ sample without Al film deposited at 700 °C, the contact angle is 165°, and the interfacial shear strength is 28 MPa. With the increase of temperature, the contact angle decreases continuously, and the interface shear strength gradually increases. The fracture of the brazed joint is transferred from the interface to the brazing seam. In comparison, the sample deposited with Al film is wetted by molten aluminum at 700 °C, and the contact angle is only 12°. The interface shear strength is about 120 MPa and is less affected by temperature. The shear fracture of the joint occurs in the brazed seam of Al metal. Therefore, the high energy generated by either the temperature increase or the particle sputtering enable the Al atoms to overcome the energy barrier to form Al–O bonds with the O atoms on the Al_2_O_3_ ceramic surface, thereby improving the wettability of Al/Al_2_O_3_.

## 1. Introduction

The wettability of the metal melt to the ceramic plays an important role in the preparation of composite materials and ceramic connection. As a typical non-reactive wetting system, there have been many research reports on the wettability of Al/Al_2_O_3_. However, due to the interference of the oxide film on the Al liquid surface, which is extremely difficult to remove, the wetting behavior is still insufficient. For example, there is a lot of controversy in the report on the change of wetting angle with temperature [1,2,3,4,5,6,7,8,9]. Some studies [5,6,7] believe that the freshly melted Al liquid cannot directly wet Al_2_O_3_, and the wetting angle is 110°–150°. As the temperature increases, the wetting angle gradually decreases, and reduces to below 90° to achieve wetting after 850 °C. There are also reports [8,9] that this wetting temperature needs to be as high as 1000–1050 °C, or even 1200 °C.

Although the above studies are controversial on the wetting transition temperature of Al/Al_2_O_3_, most of them believe that the contact angle of Al to Al_2_O_3_ ceramics will gradually decrease with the increase of the Al liquid temperature. Some studies have shown that the wettability of the Al/Al_2_O_3_ is related to the bonding state of the atoms at the interface. Zhang et al. [10] studied the interfacial structure and wetting behavior of Al and Al_2_O_3_ ceramics using molecular dynamic simulation methods. The results showed that during the heating process, as the thermal kinetic energy of atoms in Al melt increases, Al atoms gradually form Al–O chemical bonds with O atoms on the ceramic surface. The Al/Al_2_O_3_ interface also changed from the physical adsorption state to the chemical bonding state, thereby improving the wettability of Al to Al_2_O_3_ ceramics. The research of Shen et al. supports this theory experimentally [11]. They studied the wettability of Al_2_O_3_ ceramics R-type (0112), A-type (1120), C-type (0001) crystal planes and polycrystalline Al_2_O_3_ ceramics (PC) by Al melt. The results show that the order of wettability with molten Al is R ≈ A > PC > C according to the strength of the atomic bond formed with Al atoms.

However, limited by the reduction of the evaporation temperature of molten Al in a high vacuum environment, most studies on the wettability of Al/Al_2_O_3_ systems are below 1300 °C, and the lowest wetting angles obtained are also different. Some have just reached the wet state of about 90° [12,13,14], some have a better wet state of about 75° [15,16,17,18], and some may even reach about 55° [19]. The effect of higher kinetic energy of Al atoms on the wettability of Al/Al_2_O_3_ remains to be further studied. The kinetic energy of sputtered Al particles is as high as 10^0^ eV [20], which is 1–2 orders of magnitude higher than that of thermally evaporated Al. Therefore, it is expected to study the effect of higher Al atomic kinetic energy on the wettability of Al/Al_2_O_3_ using a sputtering method.

In order to reveal the effect of Al atom kinetic energy on the wettability of Al/Al_2_O_3_, two kinds of Al_2_O_3_ ceramic samples with and without Al film deposited were designed in this paper, respectively. The wetting behaviors of Al on these two Al_2_O_3_ are studied using a sealed chamber that can obtain extremely low oxygen and nitrogen partial pressure. The effect of temperature and high kinetic energy of sputtered Al particles on the wettability of Al/Al_2_O_3_ is revealed.

## 2. Materials and Methods

### 2.1. Sample Design

**Figure 1:**
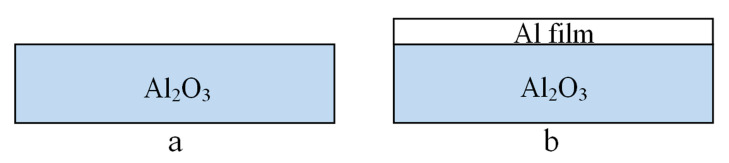
The schematic of two kinds of Al_2_O_3_ ceramic samples. (**a**) without Al film deposited; (**b**) with Al film deposited.

In order to reveal the wetting behavior of Al on Al_2_O_3_, two kinds of Al_2_O_3_ ceramic samples with and without Al film deposited on the surface were designed, respectively. The schematic diagram of the structure is shown in Figure 1. In Figure 1a, there is no film deposited on the surface of the Al_2_O_3_ ceramic sample, and a pure Al film with 1 μm thickness is deposited on the surface of the Al_2_O_3_ ceramic in Figure 1b.

### 2.2. Thin Film Deposition

The 1 mm thick polished high-purity Al_2_O_3_ ceramic substrate is ultrasonically cleaned in alcohol and placed on the substrate holder in the vacuum chamber of the multi-target magnetron sputtering system (SPC-350, Anelva, Tokyo, Japan). When the background vacuum of the vacuum chamber reaches 5.0 × 10^−4^ Pa, the ceramic substrate is baked at 400 °C × 10 min to remove the gas and impurities adsorbed on the surface. After the ceramic substrate is cooled to room temperature, Ar gas with a purity of 99.999% is filled into the vacuum chamber and the pressure is maintained at 0.6 Pa. The Ø 76 mm Al target (purity 99.99%) is controlled by a DC cathode. During the film deposition, the power of the DC cathode is maintained at 400 V × 0.5 A, and the substrate is neither heated nor applied with a negative bias. By controlling the deposition time, 1 μm thickness Al film is deposited on the Al_2_O_3_ ceramic substrate.

### 2.3. Wetting Experiment

**Figure 2:**
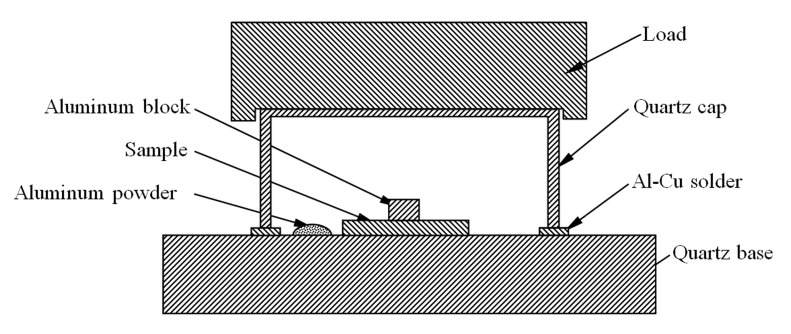
The schematic of the sealed chamber.

In order to eliminate the influence of O_2_ and N_2_ on the wetting behavior of Al after melting, the experiment adopted a sealed chamber that can cut off the oxygen and nitrogen sources. The structure of the sealed chamber is shown in Figure 2. A flat-mouthed quartz cup is buckled upside down on a quartz plate, and an aluminum-based solder plate is placed between them. The top of the quartz cup is pressed by a stainless steel weight block. When heated in a vacuum stove, the Al-Cu solder melted first and thus made the sealed chamber airtight, isolated from the outside vacuum environment. The residual O_2_ and N_2_ in the sealed chamber (including desorbed from the inner surface) would be gradually consumed due to the oxidation of the Al-Cu solder and aluminum powder. As a result, a very high vacuum was achieved.

During the wetting experiment, the Al_2_O_3_ ceramic sample with and without Al film deposited is placed in this sealed chamber. A cleaned pure aluminum block with a size of 2 × 2 × 2 mm^3^ is placed on the sample. The sealed chamber is placed in a vacuum furnace and evacuated to 10^−1^ Pa. It is heated to 700, 800, 900, 1000, and 1100 °C respectively for 30 min and then cooled with the furnace. By comparing the wetting behavior of molten aluminum on the two samples, the effect of the kinetic energy of Al atoms on the wetting of Al/Al_2_O_3_ is revealed.

### 2.4. Interface Strength Test

In our previous research, a kind of aluminum foil solder-coated bi-layer film was proposed to achieve direct brazing of ceramic without interfacial reaction layers [21]. For this paper, a 2 μm Al layer followed by a 0.1 µm Cu layer was deposited on both side of a 100 μm pure aluminum foil. When melted, the coated foil would form an Al alloy liquid with 0.1 at.% Cu. Due to the low Cu content, it can be regarded as a pure Al melt. Holding the coated Al foil between two Al_2_O_3_ substrates, a stainless steel weight was placed on top of the sample to fix and exert pressure. This brazing sample is placed flat in a vacuum furnace. When the furnace vacuum reaches 10^−4^ Pa, the Al_2_O_3_ ceramic samples are brazed at 700–950 °C for 30 min and then cooled with the furnace. The two kinds of Al_2_O_3_ ceramic samples are brazed respectively with the coated aluminum foil solder to obtain a brazed joint containing an Al/Al_2_O_3_ interface. Through the measurement of the joint shear strength and the observation of the fracture mode, the interface strength of Al/Al_2_O_3_ was studied.

**Figure 3:**
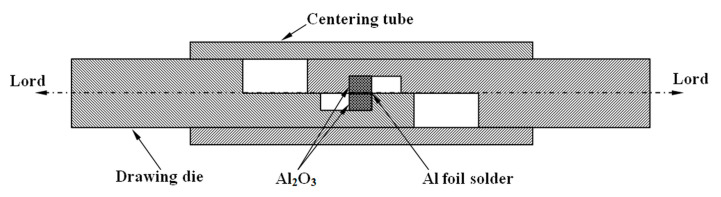
The fixture schematic diagram for measuring shear strength of brazed joints.

The structure and composition of the brazed joint are analyzed by scanning electron microscope (SEM, Hitachis-3400n, Hitachi, Tokyo, Japan) and the attached X-ray energy dispersive spectroscopy (EDS). The shear strength of the brazed joint is tested by an electronic tensile machine, and the size of the shear surface of the test sample is 3 × 2 mm^2^, where it was fixed in a special mold, as shown in Figure 3. The obtained shear strength is the average of the effective values of more than 10 samples. The morphology of the shear fracture is observed by SEM and optical microscope (OM, VHX-1000, Keyence, Osaka, Japan), whereas the composition is analyzed by EDS.

## 3. Results

### 3.1. Wetting Behavior

**Figure 4:**
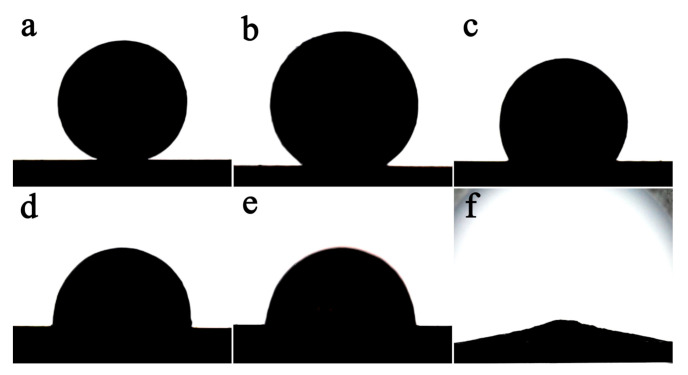
The wettability of molten aluminum on the two kinds of sample at different temperatures. Without Al film deposited sample: (**a**) 700 °C; (**b**) 800 °C; (**c**) 900 °C; (**d**) 1000 °C; (**e**) 1100 °C; with Al film deposited: (**f**) 700 °C.

Figure 4 compares the wetting behavior of molten aluminum on two samples. All the aluminum blocks were eventually clustered into a spherical shape or spread on the Al_2_O_3_ ceramic substrate. This result shows that the sealed chamber designed in this paper successfully cut off the channels for O_2_ and N_2_ to enter, and obtained a vacuum environment with extremely low oxygen and nitrogen partial pressure, preventing further thickening of the oxide film on the surface of the molten aluminum, and breaking the constraints of the surface oxide film. Therefore, the molten aluminum can flow and change shape under the action of surface tension. Figure 4a–e shows the wetting behavior of molten aluminum on samples of undeposited Al film Al_2_O_3_ at different temperatures. The molten aluminum cannot wet Al_2_O_3_ ceramics at 700 °C. This contact angle gradually decreases with the increase in temperature, and the wettability of molten aluminum to Al_2_O_3_ ceramics is improved. The contact angle decreases to a critical value of about 90° when the temperature increases to 1000 °C. With a further increase in the temperature value of 1100 °C, the wettability of molten aluminum to Al_2_O_3_ ceramics changes from non-wetting to wetting. However, the molten aluminum exhibits completely different wetting behavior on Al_2_O_3_ ceramics with Al film deposited. As shown in Figure 4f, the molten aluminum is almost completely spread on this sample at 700 °C, indicating very good wettability.

**Figure 5:**
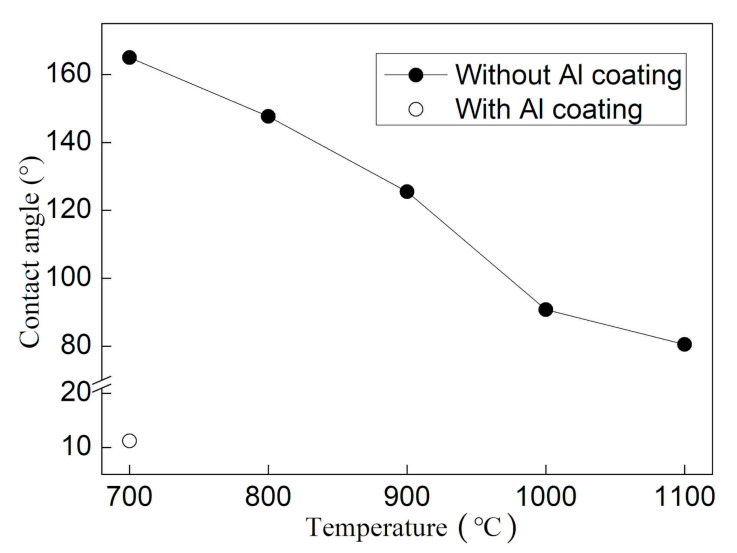
The contact angle change of molten aluminum on the two kinds of sample.

Figure 5 shows the contact angle change of molten aluminum on the two kinds of samples. For Al_2_O_3_ ceramic samples without Al film deposited, the contact angle gradually decreases with the increase in temperature, from 165° at 700 °C to 90.8° at 1000 °C. With a further increase in the temperature to 1100 °C, the contact angle decreases to 80.3°. The contact angle of molten aluminum on Al_2_O_3_ ceramics deposited with Al film is as low as 12°, at 700 °C.

### 3.2. Interface Strength

**Figure 6:**
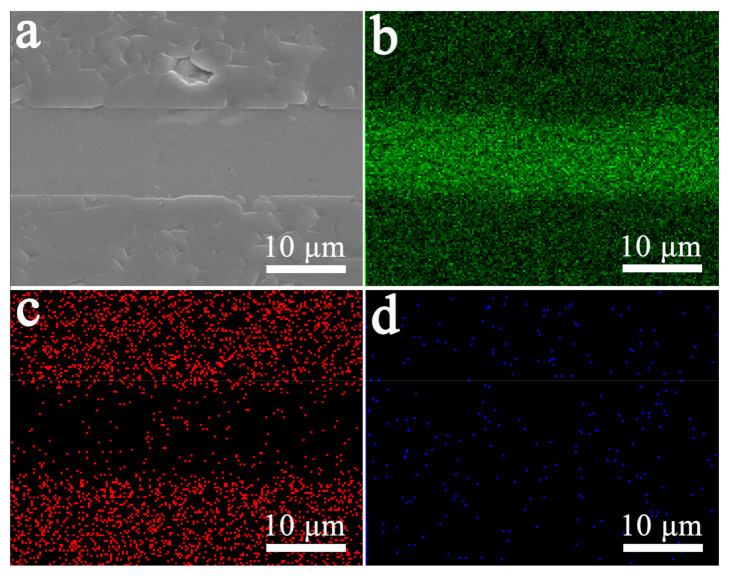
The joint SEM images of the Al_2_O_3_ ceramic sample without Al film brazed at 700 °C (**a**) and corresponding area-scan images of Al (**b**), O (**c**) and Cu (**d**) elements.

Brazing experiments show that two Al_2_O_3_ ceramic samples can be successfully brazed by Al foil solder at various temperatures. Through SEM observation, it is found that the brazed joints of the two kinds of sample obtained at different brazing temperatures have similar structures. Figure 6a shows the joint SEM morphology of the Al_2_O_3_ ceramic sample without an Al film, which is brazed at 700 °C. The Al brazing seam in this picture is dense and full, and there are no brazing defects such as incomplete penetration and pores. In addition, it can be seen from the area-scanning of Al, O, and Cu (Figure 6b–d) that the interface between the brazing seam and the Al_2_O_3_ ceramic is clear, and no element enrichment is found. This result indicates that there is not any reaction transition layer at the interface.

**Figure 7:**
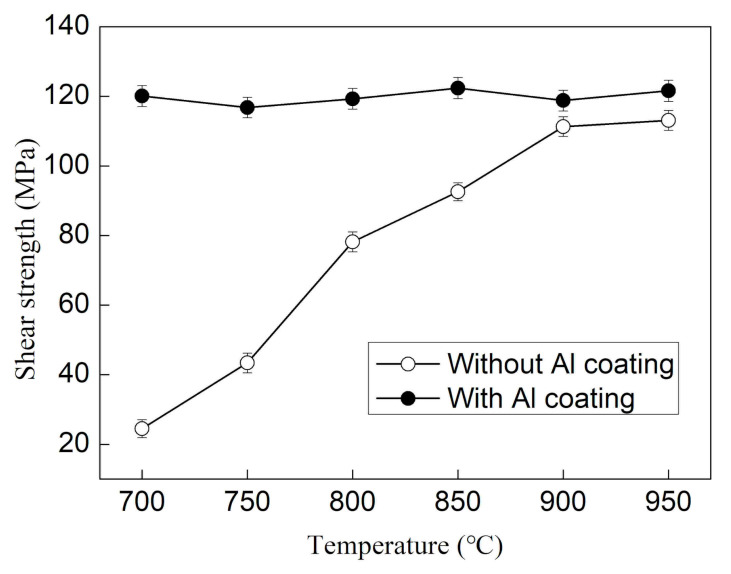
The relationship between the brazed joints shear strength of the two kinds of sample and temperature.

Figure 7 shows the relationship between the brazed joints shear strength of the two kinds of sample and temperature. For Al_2_O_3_ ceramic samples without Al film deposited, the shear strength of the joint brazed at 700 °C is 28 MPa. The contact angle gradually decreases with the increase in temperature. The shear strength of the joint increases with the increase in brazing temperature, and the increase rate slows down after reaching 115 MPa at 900 °C. It is worth noting that the shear strength of the brazed joint of the Al_2_O_3_ ceramic sample with the Al film deposited shows a significantly different trend. It has reached a high value of about 120 MPa at 700 °C, and does not change significantly with the increase in brazing temperature.

**Figure 8:**
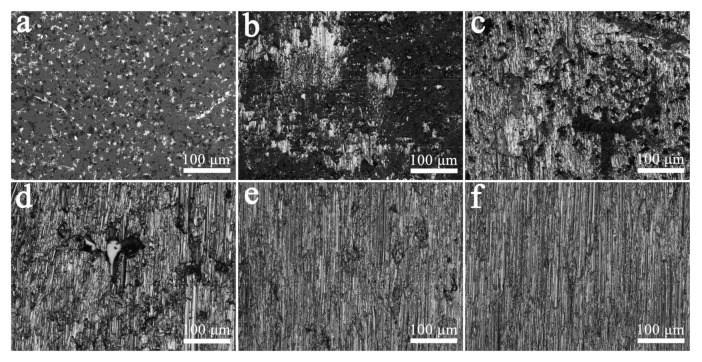
Optical microscope images of the joints fracture morphologies of the two kinds of sample brazed at different temperatures. Without Al film deposited sample: (**a**) 700 °C; (**b**) 750 °C; (**c**) 800 °C; (**d**) 850 °C; (**e**) 900 °C; with Al film deposited: (**f**) 700 °C.

The optical microscope observation of the shear fracture of each brazed joint (Figure 8) found that the fracture morphology of the Al_2_O_3_ ceramic sample without Al film deposited greatly changes with the increase in the brazing temperature. The fracture of joints brazed at 700 °C is basically on the surface of Al_2_O_3_ ceramics, and there are only a few small and scattered light-colored Al regions (Figure 8a). This shows that the fracture of this brazed joint occurs at the interface between the brazing seam and the ceramic. When the brazing temperature rises to 750 °C, the area of the light-colored Al increases (Figure 8b), indicating that partial fracture has occurred at the Al metal in the brazing seam. The area of the light-colored Al region in the 800 °C joint fracture continues to increase, and the furrow-like morphology along the direction of the shear force formed after the ductile Al shear fracture is observed (Figure 8c). When the brazing temperature is increased to 900 °C, the fracture of the joint is entirely composed of the furrow-like morphology of Al metal (Figure 8e). This series of changes shows that the shear fracture of the joint gradually shifts from the interface to the brazing seam as the temperature increases. Combining the wettability results in Figure 4 and the shear strength of the joint in Figure 7, the increase in the shear strength with the brazing temperature comes from the improvement of the wettability of Al_2_O_3_ ceramics by molten aluminum, which increases the interfacial strength of Al/Al_2_O_3_.

Figure 8f shows the shear fracture morphology of the Al_2_O_3_ joint with Al film at 700 °C. Different from the Al_2_O_3_ joint without Al film, this fracture morphology is composed of furrow-like morphology and does not change significantly with the increase in temperature. This result is also consistent with the joint shear strength in Figure 7. The joint shear strength has reached a high value of about 120 MPa at 700 °C, and does not change significantly with the increase in brazing temperature. Further combining the wettability results in Figure 4, it can be concluded that the Al film directly sputtered and deposited on the Al_2_O_3_ ceramic has the effect of improving the wettability and interface strength.

**Figure 9:**
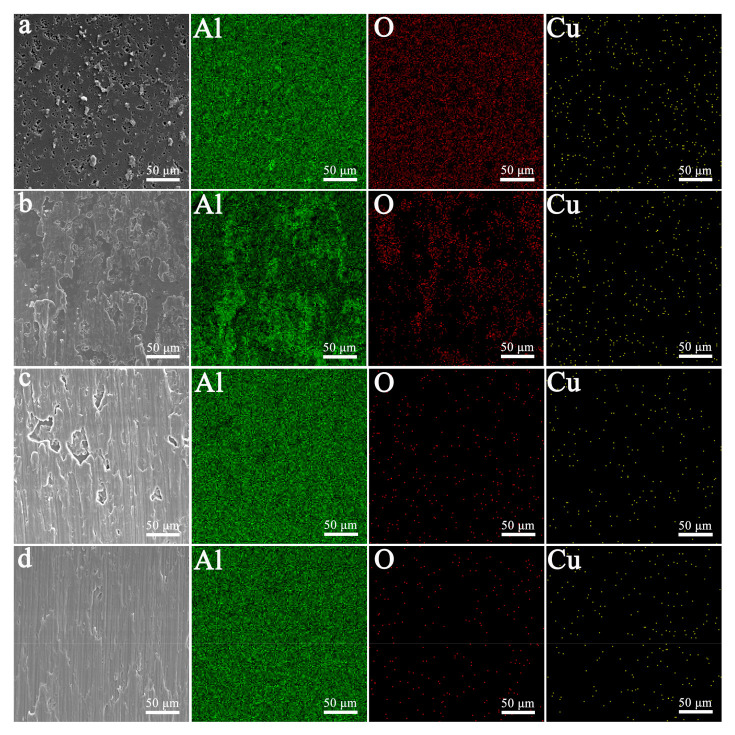
The area-scanning results of the Al, O, and Cu elements of the brazing fractures of the two kinds of sample. Without the Al film deposited sample: (**a**) 700 °C; (**b**) 800 °C; (**c**) 900 °C; with Al film deposited: (**d**) 700 °C.

Figure 9 shows the area-scanning results of the Al, O, and Cu elements of the brazing fractures of the two kinds of sample. A large amount of O elements and very few Al-rich regions are distributed in the fracture of the undeposited Al film samples at 700 °C (Figure 9a). This result indicates that the fracture mainly occurs at the interface between the brazing seam and the ceramic. As the brazing temperature increases, the area of the Al-rich area on the fracture gradually increases, and the corresponding O element decreases. When the temperature increased to 900 °C, the fracture was mainly covered by Al element (Figure 9c), indicating that the fracture mainly occurred in the Al brazing seam. For the sample deposited with the Al film, the fracture at 700 °C is basically covered by Al (Figure 9d), which indicates that the fracture of the joint at this temperature has occurred in the Al weld.

## 4. Discussion

The study of undeposited Al film samples in this article shows the effect of temperature on the wettability of Al/Al_2_O_3_. With the increase in the system temperature, the contact angle of molten aluminum to Al_2_O_3_ decreases continuously, the Al/Al_2_O_3_ interface strength gradually increases, and the shear fracture of the joint shifts from the interface to the brazing seam. The improvement of the wetting state mainly comes from the decrease in the liquid/solid interfacial tension of the system, which is essentially the Al–O chemical bond gradually formed between the Al atom in the molten aluminum and the O atom on the surface of Al_2_O_3_. According to the molecular dynamics study of Zhang et al. [10] and the first-principles study of Sun et al. [22], there are two binding states between the molten Al atom and the O atom on the ceramic surface. When the temperature of the system is low, physical adsorption with “gap” is formed between them, and as the temperature increases, a chemical bond with an Al–O chemical bond is formed. The formation of the Al–O chemical bond significantly reduces the interfacial tension between molten aluminum and Al_2_O_3_, which makes molten aluminum wetting Al_2_O_3_. The increase in temperature provides kinetic energy for Al atoms to overcome the barrier that forms the Al–O bond. It should be noted that wetting is a progressive process, because the formation of Al–O bonds is not only related to the kinetic energy of Al atoms, but is also related to the existence state of Al_2_O_3_ terminal atoms. Pilania’s research [23] shows that the contact angle of molten Al on different crystal planes of Al_2_O_3_ is different at the same temperature.

The study of deposited Al film samples shows that the sputtering deposition of Al film can improve the wettability of Al/Al_2_O_3_. During magnetron sputtering, the kinetic energy of gas phase particles (atoms or ions) sputtered from the cathode target surface is as high as 10^0^ eV [20]. This energy is not only much higher than the thermal kinetic energy of molten aluminum atoms, but even an order of magnitude higher than the energy of Al atoms when they are thermally evaporated above 1000 °C. The impact of these high-energy sputtered Al particles during deposition is sufficient to overcome the energy barrier and form Al–O chemical bonds with O atoms on the Al_2_O_3_ surface. Moreover, once these Al–O chemical bonds are formed, they can still be maintained after the Al film is melted. In this case, the molten aluminum can wet the ceramic at the just melting temperature. Therefore, although the sputtered Al film is solid, from the point of view of forming Al–O bonds, these Al films have “wetted” Al_2_O_3_. Therefore, despite the solid state, these Al films have “wetted” Al_2_O_3_ from the perspective of forming Al–O bonds. According to the above analysis, as long as the energy is high enough, Al atoms can form Al–O chemical bonds with the O atoms on Al_2_O_3_ regardless of whether the interface is liquid/solid or solid/solid.

## 5. Conclusions

In this paper, two kinds of Al_2_O_3_ ceramic samples with and without Al film deposited were designed respectively. The influences of temperature and high kinetic energy sputtering particles on the wettability and interface strength of Al/Al_2_O_3_ were studied by comparing the wetting behavior of molten aluminum on the surface of two samples. The effect of the kinetic energy of Al atoms on the wettability of Al/Al_2_O_3_ was revealed.

1. For the sample without Al film deposited, molten aluminum does not wet Al_2_O_3_ at 700 °C, the contact angle is 165°, and the interfacial shear strength is 25 MPa. With the increase in temperature, the contact angle decreases continuously, and the interface shear strength gradually increases. The contact angle decreases to a critical value of about 90° when the temperature increases to 1000 °C. With further increases in the temperature value of 1100 °C, the contact angle decreases to 80.3°. The wettability of molten aluminum to Al_2_O_3_ ceramics changes from non-wetting to wetting. The shear strength of the joint gradually increases to 115 MPa at 900 °C, and then the increase slowed down.

2. The sample deposited with Al film is wetted by molten aluminum at 700 °C, and the contact angle is only 12°. The interface shear strength is as high as about 120 MPa and is less affected by temperature. The shear fracture of the brazed joint occurs in the brazed seam of Al metal.

3. The high energy generated by either the temperature increase or the particle sputtering enables the Al atoms to overcome the energy barrier to form Al–O bonds with the O atoms on the Al_2_O_3_ ceramic surface. The formation of Al–O chemical bonds is the fundamental reason for the improvement of Al/Al_2_O_3_ wettability.

## Data Availability

The data presented in this study are available on request from the corresponding author.

## References

[1] Ma S., Yan R., Zong N., Davidchack R.L., Dong H. (2020). Unveiling the influence of interfacial bonding and dynamics on solid/liquid interfacial structures: An ab initio molecular dynamics study of (0001) sapphire-liquid Al interfaces. Phys. Rev. Mater..

[2] Fang C., Yasmin S., Fan Z. (2021). Interfacial interaction and pre-nucleation at liquid-Al/γ-Al_2_O_3_{1 1 1} interfaces. J. Phys. Commun..

[3] Voigt C., Ditscherlein L., Werzner E., Zienert T., Nowak R., Peuker U., Sobczak N., Aneziris C.G. (2018). Wettability of AlSi7Mg alloy on alumina, spinel, mullite and rutile and its influence on the aluminum melt filtration efficiency. Mater. Des..

[4] Schneider G., Marta F., Weber L., Mortensen A. (2020). Kinetic processes in the high-temperature pressure-infiltration of Al into Al_2_O_3_. Acta Mater..

[5] Ghosh S., Chakraborty R., Dandapat N., Pal N.S., Datta S., Asu D.B. (2012). Characterization of alumina–alumina/graphite/monel superalloy brazed joints. Ceram. Int..

[6] Sangghaleh A., Halali M. (2008). An investigation on the wetting of polycrystalline alumina by aluminium. J. Mater. Process. Technol..

[7] Xia H., Wu A., Fan F., Zou G., Ren J. (2012). Effects of ion implantation on the brazing properties of high purity alumina. Surf. Coat. Technol..

[8] Lee S.B., Kim Y.M. (2011). Direct observation of in-plane ordering in the liquid at a liquid Al/α-Al_2_O_3_ interface. Acta Mater..

[9] Yang J., Bao S., Akhtar S., Shen P., Li Y. (2020). Influence of grain refiners on the wettability of Al_2_O_3_ substrate by aluminum melt. Metall. Mater. Trans. B.

[10] Zhang Q., Cagin T., Duin A.V., Iii W.A.G., Yue Q., Hector L.G. (2004). Adhesion and nonwetting-wetting transition in the Al/α-Al_2_O_3_ interface. Phys. Rev. B.

[11] Shen P., Fujii H., Matsumoto T., Nogi K. (2003). The influence of surface structure on wetting of α-Al_2_O_3_ by aluminum in a reduced atmosphere. Acta Mater..

[12] Aguilar-Santillan J. (2009). Wetting of Al/sapphire (0001) system: Measurement effect and affecting factors. Metall. Mater. Trans. B.

[13] Klinter A.J., Suarez G.M., Drew R.A.L. (2008). Wetting of pure aluminum and selected alloys on polycrystalline alumina and sapphire. Mater. Sci. Eng. A.

[14] Levi G., Kaplan W.D. (2002). Oxygen induced interfacial phenomena during wetting of alumina by liquid aluminium. Acta Mater..

[15] Brennan J.J., Pask J.A. (1968). Effect of nature of surfaces on wetting of sapphire by liquid aluminum. J. Am. Ceram. Soc..

[16] Jung W., Song H., Sang W.P., Kim D.Y. (1996). Variation of contact angles with temperature and time in the Al-Al_2_O_3_ system. Metall. Mater. Trans. B.

[17] Naidich Y.V., Chubashov Y.N., Ishchuk N.F., Krasovskii V.P. (1983). Wetting of some nonmetallic materials by aluminum. Powder Metal. Met. Ceram..

[18] Ping S., Fujii H., Matsumoto T., Nogi K. (2004). Critical factors affecting the wettability of α-Alumina by molten aluminum. J. Am. Ceram. Soc..

[19] Bao S., Tang K., Kvithyld A., Tangstad M., Engh T.A. (2011). Wettability of aluminum on alumina. Metall. Mater. Trans. B.

[20] Zheng W.T., Ding S.L. (2004). Physical methods of film preparation. Thin Film Materials and Thin Film Technology.

[21] Zhang X.Y., Ma B.Y., Li R.B., Li G.Y. (2018). Brazing of coated Al foil filler to AlN ceramic. Acta Metall. Sin..

[22] Sun S.Y., Xu P.P., Ma B.Y., Shang H.L., Li G.Y. (2019). Effects of temperature and O partial pressure on the atomic structure of Al_2_O_3_ (0001) surface. Comput. Mater. Sci..

[23] Pilania G., Thijsse B.J., Hoagland R.G., Lazic I., Liu X.Y. (2014). Revisiting the Al/Al_2_O_3_ interface: Coherent interfaces and misfit accommodation. Sci. Rep..

